# The right to health, health systems development and public health policy challenges in Chad

**DOI:** 10.1186/s13010-015-0023-z

**Published:** 2015-02-15

**Authors:** Jacquineau Azétsop, Michael Ochieng

**Affiliations:** Département de Santé Publique, Faculté des Sciences de la Santé de l’Université de N’djaména, Avenue Mobutu, BP 1117, N’ djaména, Tchad; Georgetown Jesuit Community, PO Box 571200, Washington, DC 20057 USA

**Keywords:** The right to health, Health systems development, Social justice, Health policy, Intersectoral collaboration, Public health and democratic governance, Community agency, Social participation, Accountability and transparency

## Abstract

**Background:**

There is increasing consensus that the right to health can provide ethical, policy and practical groundings for health systems development. The goals of the right to health are congruent with those of health systems development, which are about strengthening health promotion organizations and actions so as to improve public health. The poor shape and performance of health systems in Chad question the extent of realization of the right to health. Due to its comprehensiveness and inclusiveness, the right to health has the potential of being an organizational and a normative backbone for public health policy and practice. It can then be understood and studied as an integral component of health systems development.

**Method:**

This paper uses a secondary data analysis of existing documents by the Ministry of Public Health, Institut National de la Statistique, des Etudes Economiques et Démographiques (INSEED), the Ministry of Economy and Agence Française de Cooperation to analyze critically the shape and performance of health systems in Chad based on key concepts and components of the right to health contained in article 12 of the International Covenant on Economic, Social and Cultural Rights, and on General Comment 14.

**Results:**

The non-realization of the right to health, even in a consistently progressive manner, raises concerns about the political commitment of state officials to public health, about the justice of social institutions in ensuring social well-being and about individual and public values that shape decision-making processes. Social justice, democratic rule, transparency, accountability and subsidiarity are important groundings for ensuring community participation in public affairs and for monitoring the performance of public institutions.

**Conclusion:**

The normative ideals of health systems development are essentially democratic in nature and are rooted in human rights and in ethical principles of human dignity, equality, non-discrimination and social justice. These ideals are grounded in an integrated vision of society as a place for multi-level interactions, where government plays its role by equitably providing institutions and services that ensure people’s welfare. Inter-sectoral collaboration, which calls for a conceptual shift in health and public policy, can be instrumental in improving health systems through concerted efforts of various governmental institutions and civil society.

## Introduction

In common with most modern human rights, the right to health (RH) is comprehensive and morally compelling, for it does not simply uncover empirical facts of systemic violations of human dignity but points to the messiness of the sociopolitical arena and establishes the normative obligations of the institutional bodies that have the human rights responsibility to protect population health. Properly understood, the RH has a profound contribution to make toward building healthy societies and equitable HS. This right is grounded in a broad definition of health as “a state of complete physical, social and mental well-being, and not merely the absence of disease or infirmity” [1]. Health is regarded by the World Health Organization (WHO) as a fundamental human right and, correspondingly, WHO holds that all people should have access to basic resources for health. Health is a resource which allows people to lead a productive life because its acquisition conditions access to many goods. Hence, “Arguments to do with a right to health are therefore concerned with claims to live in a physical and social environment that does not prejudice the prospects for leading a full and healthy life, including access to health services” [2]. These arguments raise concern about the justice of social institutions in providing access to healthcare to all and ensuring people’s well-being. The goals of the RH are congruent with those of health systems development (HSD), which is about strengthening health promotion organizations and actions so as to improve public health.

The normative ideals of HSD are essentially democratic, rooted in human rights and ethical principles of human dignity, equality, non-discrimination and social justice. These democratic ideals are partly prompted by the inclusiveness of the RH, which presupposes an understanding of health as a good connected to other social goods. Our approach to the justice of HSD will logically demand equity in all the domains that interface with human health. This approach to justice is rooted in an integrated vision of society as a place for multi-level interactions, where government plays its welfare-enhancing role through an equity-based provision of public services and dimensions of well-being [3]. Inter-sectoral collaboration is a way to implement our approach to health justice. It calls for a conceptual shift in health and public policy. Inter-sectoral collaboration offers a good prospect for an integrated response to well-being issues by the government. Democratic transparency, accountability and subsidiarity are important groundings for ensuring the public’s participation in society’s affairs and monitoring the performance of public institutions.

## Method

This paper uses a secondary data analysis of existing documents on HS by the Ministry of Public Health (MPH), Institut National de la Statistique, des Etudes Economiques et Démographiques (INSEED), Ministry of Economy and Agence Française de Cooperation to analyze critically the shape and performance of health systems (HS) in Chad. As hermeneutical tools, this critical analysis uses key concepts and components of the right to health contained in article 12 of the International Covenant on Economic, Social and Cultural Rights and in General Comment 14. From Article 12, the performance of health systems are evaluated on four main points: the state of infant and maternal health, environmental conditions, disease prevention and access to primary care. From General Comment 14, the paper derives the core obligations of the state and criteria for evaluating the progressive realization of the RH by state’s institutions. The paper strategically uses the right-to-health analysis of HS, an element of the entire social system, to point out important values and policies that may improve the responsiveness of the healthcare system to people’s needs.

### The right to health in international primary healthcare and human rights documents

The recognition of the RH prescribed by the Universal Declaration of Health (UDHR) in 1948 was further confirmed by the 1978 Alma-Ata Declaration on primary health care (PHC) and subsequent PHC documents. Health promotion, one of the fundamental aspects of PHC, has been addressed by the Ottawa Charter (1986) and subsequent health promotion documents from Adelaide (1988), Sundsvall (1991), Jakarta (1997), Mexico (2000), Bangkok (2005) and Nairobi (2009). The RH is also recognized in major human rights instruments, including the UDHR (Art 25 (1)), the 1965 International Convention on the Elimination of All Forms of Racial Discrimination (art. 5), ratified by the Republic of Chad in August 1997; the 1966 International Covenant on Economic, Social and Cultural Rights (art. 12), ratified in June 1995; the 1979 Convention on the Elimination of All Forms of Discrimination Against Women (arts. 11 (1) (*f*), 12 and 14 (2) (*b*)), ratified in June 1995; the 1989 Convention on the Rights of the Child (art. 23, 24), ratified in September 1990; the 1990 International Convention on the Protection of the Rights of All Migrant Workers and Members of Their Families (arts. 28, 43 (*e*) and 45 (*c*)), signed in September 2012; and the 2006 Convention on the Rights of Persons with Disabilities (art. 25), signed in September 2012. Numerous conferences and declarations endorsed by the Republic of Chad, such as the International Conference on PHC, the United Nations Millennium Declaration and Millennium Development Goals and the Declaration of Commitment on HIV/AIDS have also helped clarify various aspects of public health relevant to the RH and have reaffirmed commitments to its realization. The RH is also recognized in the African Charter on Human and Peoples’ Rights (1981), which was ratified by the Republic of Chad in October 1986.

The RH is a constitutionally entrenched right which is acknowledged in Article 18 and recognized as a social right in article 17 of the Constitution. Article 26 emphasizes the state’s obligation to fulfill this right [4]. The RH is one of the guiding principles of the National Health Policy (NHP), of Law 006/PR/2002 on reproductive health [5], of Law 19/PR/2007 on the protection of people living with HIV from discrimination [6] and of many other health laws. The penal code in articles 27 and 244 ensures the right to life and physical integrity without which the RH is meaningless [7].

The formulation of the RH varies from one document to another. The formulation contained in article 12 of the International Covenant on Economic, Social and Cultural Rights highlights and recognizes the RH in its first paragraph. The second paragraph outlines areas in which governments ought to guarantee full realization of the RH: first, reducing infant mortality and providing for the healthy development of children; second, improving environmental conditions and closer monitoring of the consequences and working conditions of industry; third, disease prevention, treatment and monitoring, including preventive HS and systems for monitoring occupational health; and, fourth, providing basic medical services for the entire population [8]. General Comment 14 outlines the core obligations that the state should fulfill to achieve the RH. In addition, it points to the obligatory services and activities that each state should progressively place at the disposition of people. The services are the same as those mentioned in General Comment 12. The obligations include the recognition of the RH, the provision of remedies for redress, and the obligation of the state to refrain from laws, policies and activities that impede the realization of these rights. The realization of the RH by states is not an option, but an obligation required by international law. We do not mean that this right can be realized by a poor state like Chad overnight. The right to health is subject both to progressive realization and resource availability. Although expressed in this way, nonetheless, the RH imposes technical and legal obligations of immediate effects. The technical obligations have been fulfilled to some extent. These obligations include preparing a national plan for health care and protection, designing indicators and benchmarks for monitoring progress and encouraging individual and community participation in health decisions. The legal obligations refer to the need for addressing the rights to underlying determinants of health (water, food, housing, access to information and sanitation). In spite of efforts deployed by subsequent governments to strengthen HS, the country has not being able to improve health indicators due to poor management of scarce resources and the lack of professionalism and public values that can sustain the work of the healthcare system. With current HS, realizing the RH can only be a dream if the entire social system is not improved.

The RH does prescribe unlimited access to healthcare services and resources that ensure well-being. It does not mean the right to be healthy, for it “takes into account individual’s biological and socio-economic preconditions and a state’s available resources” [9]. Some aspects of health, including the individual’s proneness to disease and genetic factors, are beyond the scope of what the state can do. The RH would be better understood as a right to health protection, including two components: a right to health care and a right to healthy conditions. International human rights law links the right to health to other human rights, including the rights to food, housing, work, education and social protection. Good health is determined not only by preventing and treating disease, but also by many other aspects of development.

### Poor health indicators, a sign of health systems failure

HS performance in Chad is quite low, as indicators of infant and maternal mortality, access to basic care, disease prevention and epidemiologic surveillance show. Poor health indicators reflect, partly, the actual state of HS. With fragmented, understaffed, underfunded and poorly coordinated HS, significant improvement in public health cannot be achieved.

#### Access to care, maternal health and child healthcare

In Chad, access to care is provided through four main mechanisms: direct payment, free access to selected services, health insurance and health mutual. The out-of-pocket payment is the most common mechanism of healthcare financing, as it represents about 50% of total health expenditure. Free health care concerns emergency surgery, obstetric and medical care. Financed entirely by the state with the support of its partners, this measure was introduced in hospitals in 2008 as part of the new social policy by the head of state. Other measures of gratuity are applied to selected diseases (chronic malaria, AIDS, tuberculosis, etc.) and specific population groups such people living with HIV, under-five children and pregnant women. However, these measures are not accompanied by compensatory mechanisms that reduce their negative effects on the already underfunded healthcare systems (HCS). Health insurance is used by less than 2% of people. It is usually contracted by large corporations for the benefit of their employees. Health mutuals are currently in their experimental phase in the southern regions [10]. In spite of the efforts deployed by the state to improve health status in Chad, access to basic care remains a major challenge to most people, due to socioeconomic and geographical reasons. To access care, patients travel an average of 14.4 kilometers [11]. Complementary strategies to provide hard-to-reach and marginalized population groups with adequate health services and infrastructures are poorly developed or non-existent. To avoid the negative effects of impoverishing out-of-pocket expenditures on households, the state should develop social protection mechanisms, including social health insurance and tax-funded systems.

A minimal essential package of health-service facilities is prescribed by the National Health Policy, which includes the Minimal Package of Activities (MPA) for integrated healthcare centers and the Complementary Package of Activities (CPA) for district hospitals. Each healthcare center is organized in areas of responsibility. The realization of MPA and CPA remains low. Out of 1,305 areas of responsibility, only 1,061 are functional. This corresponds theoretically to a coverage rate of 81.30% [12]. This rate is theoretical because MPA is not fully delivered in most healthcare centers.

The HCS has not reduced the rate of maternal mortality in the last 15 years. This rate has increased, reaching 1,086 deaths per 100,000 live births in 2012 against 827 in 1997 [11], while several countries in sub-Saharan Africa have reduced the rate by half between 1990 and 2010. Many reasons account for this high rate, one of them being the low rate of births attended by a health professional. The rate of births attended by a health professional was 22.7% in 2012 against 16.2% in 2000, resulting in an increase of 6.5% in 12 years [12]. On the other hand, the rate of postnatal consultations has been less than 4% since 2000. Even though the use of antenatal care (ANC) has increased, the rate remains the lowest in Africa. Still in 2012, nearly 50% of women had no access to ANC during pregnancy. This proportion exceeds 67% in the regions of Batha, Salamat and Wadi Fira. Less than 25% of women (50% in N’djaména) completed the four ANC visits recommended by the WHO [10].

Just as for maternal health, infant mortality rates are quite high. One in every six children in Chad dies before the age of five, and citizens of Chad can expect to live for no longer than 50 years [12]. Approximately 65% of infants’ deaths occur because of all sorts of diseases. Malnutrition is responsible for more than half of under-five deaths, which occur in the context of lack of preventive and primary health care, including maternal and child health care. In addition to these causes, only 3% of children are exclusively breastfed till 6 months of age. As a result, 39% of under-five children suffer from chronic malnutrition and 21% suffer from severe malnutrition [13]. Child malnutrition begins in the womb, as many mothers do not have enough to eat and lack adequate ANC. More than 20% of women suffer from acute malnutrition [11]. The utilization rate of infant preventive consultation was 24.97% in 2012, suggesting a low ability to detect health threats, including malnutrition. Similarly, the under-five children immunization coverage remains low, suggesting HCS’s failure to reduce infant mortality and morbidity from preventable diseases. On average, only 8% of children aged 12–23 months are completely vaccinated against targeted childhood diseases, while 33% have never been given a single vaccine [11].

#### Social conditions, epidemiologic surveillance and health information systems

Chad is a large country (1,284,000 sq km), a landlocked nation located in the heart of sub-Saharan Africa, with a population of 12,240,127, of which 21.9% is urban. The Human Development Index in 2011 was 0.328. More than 50% of people live below the poverty threshold [12]. The low literacy rate limits the use of health services. Illiteracy limits people’s ability to have control over their health. The epidemiological profile of Chad is characterized by the prevalence of endemic and epidemic diseases, foremost among them malaria, tuberculosis, acute respiratory infections, HIV and AIDS and diarrhea [14]. Some non-communicable conditions, such as malnutrition, trauma, and cardiovascular and metabolic diseases, are also important causes of morbidity and mortality [12]. Most of the time, priority is given to emergencies and immediate injuries or illnesses, neglecting prevention. The actual state of the epidemic surveillance system betrays a lack of commitment to population health. The human cost of this inertia is simply unacceptable, when we know that some of the endemic pathologies that have claimed many lives are preventable.

The living conditions are unsanitary: only 15% of people used toilets in 2010; garbage and wastewater disposal is almost nonexistent; access to clean water remains low, with only about 52% using an improved water source [13]. Reducing vulnerability to environmental conditions is necessary to create conditions conducive to well-being. To ensure safety, an integrated policy approach, which includes determinants of health, can be designed to address the inter-linked health and environment issues [15].

The healthcare system also lacks trained public health practitioners and health statisticians to organize public health outreach programs and to gather health data. The country has a number of midwifery schools in place, but gaps in health management and information systems make it difficult to predict the number of midwives the country will have in the coming years. The lack of sound data deprives policy-makers of empirical and evidentiary proofs needed for improving HS performance. The lack of data is due not to the absence of tools or platforms for information, but to the glaring lack of national capacity to implement and maintain these tools. The lack of epidemiologists limits the extent of epidemiological research and intervention. Furthermore, epidemiological surveillance is essentially passive. It is limited to the reporting cases of diseases under surveillance from the district to the region and then to the Ministry of Public Health.

#### Understaffed and underfunded health systems

The RH cannot be realized with poorly resourced, fragmented and underfunded HS. In 2012, there was one doctor for 28,466 inhabitants in Chad, instead of 1 per 10,000 population, as recommended by the WHO; one advanced nurse for 12,903 inhabitants; one midwife for 9,596 women of childbearing age; and one qualified nurse (any category) for 2442 inhabitants [16]. For example in Bokoro, the main city of the region of Hadjer Lamis, there is only one doctor at the regional hospital for a population of 244,000. The region’s 13 health centres are poorly equipped and are staffed with one nurse for several thousand people. The situation in Bokoro depicts what goes on in most cities of the country. The shortage of specialists is felt at all levels of the HCS and all over the country. Some key specialties are sorely lacking: only 16 obstetricians and one single anesthetist were active in the public sector in 2011 [17].

Due to the shortage of physicians, health district commissioners are often involved in clinical work, besides ensuring oversight of activities in the district. The accumulation of positions by one person causes an overload of work, which is detrimental to the quality of health services. The extent of human resource shortage precludes significant improvement in population health. In addition, there is a decline in human resources, with a high rate of turnover of physicians from the public to the private sector due to poor remuneration in the former. The Ministry of Public Health (MPH) needs to improve staff motivation and retention in order to enhance the coverage and quality of care in the country.

The healthcare system (HCS) is not only understaffed but also underfunded. Health financing aims “to make funding available, ensure rational selection and purchase of cost effective interventions, give appropriate financial incentives to providers, and ensure that all individuals have access to effective health services” [18]. The insufficiency of financial resources also limits HS performance. Despite repeated commitments from the government to allocate 15% of the national budget to the health sector, this percentage steadily increased from 13 to 14% between 2000 and 2005 and decreased by 3% between 2008 and 2010. The MPH budget represented 6.47% of the national budget in 2011 (€149 million) and 5.67% in 2012 (€128 million) [12]. This reduced funding hardly reaches Regional Delegations. Towards the end of 2012, the state committed to increase the resources devoted to health. In 2013, the estimated budget represented 9.8% of the national budget (€198 million). In addition to financial struggles, the HCS is poorly equipped with basic amenities for acceptable functioning. More than 50% of healthcare centers are not equipped with electricity and almost 25% have no access to water [10]. Most of these institutions lack transportation means, operating theaters and surgical equipment.

### Contribution of the right to health to health systems development

What does a RH analysis bring to health systems development (HSD)? How can the RH provide both tools for adequate leadership and an integrated vision to improve population health in Chad? Jonathan Mann appreciated the language of modern human rights because of its integral comprehensiveness and moral urgency. That is, human rights language could link global campaigns for the right to access to medical treatment with equally effective and strategic movements to obtain greater equality in political, economic and social forms of life [19]. Mann actively promoted the idea that health and human rights are integrally connected, arguing that these fields overlap in their respective philosophies and objectives to improve well-being and to prevent premature death [20]. To guide this relationship, Mann and colleagues suggested a three-part framework [21]. Mann later added a fourth one, referring to human rights as the conscience of public health [19,22]. The fourth approach integrates the three others, showing how the impacts of health policy on health, the health consequences of human rights violations and the mutual promotion of health and human rights all have ethical implications for public health. Focusing on the fourth part of Mann’s framework, we endeavor to demonstrate that the RH can be understood as necessarily integral to the ethics of HSD in Chad, a country where the weaknesses of HS undermine access to curative and preventive care for all.

The immediate consequence of applying the RH to HSD is that the state must be deeply involved in and ultimately responsible for ensuring that its poorest citizens have access to health care. Civil society and human rights activists need to build a strong RH-movement. By doing so, “Not only can the politics of exclusion and the economics of inequity be overcome, but effective healthcare systems can be developed and high-quality health care delivered to the…poor” [23]. The government should normally be held accountable when the HCS fails to take effective steps progressively to improve HS and to tackle health inequities. As a public health initiative, HSD is primarily an ethical and political endeavor whose fundamental goal is to promote human welfare by improving each component of HS and by placing the concerns of the marginalized at the centre of health policy.

The RH is not a mere slogan for public health advocacy. It imposes an obligation on state actors, such as non-discrimination, equality, equity and a national plan for harm reduction and health promotion [24]. Achieving this right requires that there are indicators and benchmarks to monitor its progressive realization and that individuals and communities have opportunities for active and informed participation in health decision making that affects them. Because the right to health gives rise to legal entitlements and obligations, effective mechanisms of monitoring and accountability that ensure effective realization are needed [25]. Beside this more constructive role, the RH can be seen as a denunciatory and critical tool. The MPH has developed a comprehensive National Health Policy (NHP). However primary healthcare (PHC) service delivery and NHP implementation have not reached the expected standard. A RH analysis can help identify and expose organizational disorder, technical mistakes, ethical misconduct, lack of competence and immoral practices that sustain HS failures. There is a growing consensus that a good healthcare system (HCS) “is an essential element of a healthy and equitable society” [24]. However, in Chad, the performances of HCS point to failing and fragmented HS. Sustained development and population health improvement cannot be achieved with HS which are inequitable, regressive and unsafe [24].

### Inclusiveness of the right to health and inter-sectoral collaboration

Mann argued that “human rights offer a societal-level framework for identifying and responding to the underlying societal determinants of health” [19]. The RH is an inclusive right because, as a social good, health is a sphere of justice connected to other spheres of social life. The RH provides a framework for analyzing the fragmentation of health policy with coherent conceptual categories. The RH demands an effective coordination between “various sectors and departments, such as health, environment, water, sanitation, education, food, shelter, finance, and transport. The realization of this right also demands coordination within ministerial sectors and between governmental departments. The need for coordination extends to policy-making and the actual delivery of services” [26]. Otherwise the HCS will be improved, but other rights whose fulfillment contributes to people’s well-being will not be promoted.

More and more, the need for inter-sectoral collaboration appears to be a moral imperative rooted in the intrinsic interdependence that exists between the RH and other rights contained in the Universal Declaration Human Rights. Human rights are interdependent, indivisible and interrelated. The violation of the RH may often impair the enjoyment of other human rights. The importance given to the underlying determinants of health, that is to the factors and conditions that protect and promote the RH beyond health services and facilities, shows that the RH is dependent on, and contributes to, the realization of many other human rights. Policy that aims at promoting health cannot focus solely on healthcare provision but should include these underlying determinants without which the RH cannot be realized. Thus, the realization of the RH demands attention to the increasingly complex relationship of people to their environment. This has implications for training in medical schools. It is no longer enough to train future physicians in clinical and preventive interventions, but it has become necessary also to provide them with skills in contextualizing sciences (medical humanities, medical anthropology, and political and social sciences) so that they may be well-equipped to understand and address social forces that shape risks for disease. The inclusive nature of the RH demands a policy shift.

The Chadian government needs to commit several other sectors toward a common goal of improving public health and living conditions. This shift requires a general policy review to align all sectors toward a common vision. The health sector can play a major supporting role in creating a platform for inter-sectoral collaboration. A change of policy culture is needed to ensure that health professionals work beyond their traditional sector to promote the RH. As a social institution, healthcare system (HCS) depends upon other social institutions. The failures of the HCS are often determined by failures in other sectors.

### Democratic governance and health policy

The inclusiveness of the RH suggests that determinants of health are not purely biological but are also social and political in nature. The RH perspective is consistently compatible with work in social epidemiology that has established social determinants as distal causal factors of disease [27]. Social determinants of health are influenced in many ways by forms of discrimination found in society. For example, rural dwellers and the urban poor who already suffer socioeconomic exclusion are more likely to lack access to care than others. Issues of access to care cannot be resolved within the HCS in isolation from the broader social context but, more fundamentally, ought to be approached as democratic and social justice issues. Framing health systems development (HSD) as a challenge critical to the realization of the RH provides groundings for linking public health with social justice and democratic ideals, because it brings to the forefront the ability of HCS and other social institutions to address justly and efficiently the macro-determinants of health. The ever growing literature in social epidemiology that provides the theoretical and empirical justification for connecting public health and the construction of democratic society can be utilized to substantiate this link [28]. This is done by examining not just the impacts of resource allocation and distribution on population health outcomes but also by studying resource allocation and distribution in relation to policies, legal arrangements and public practices that determine both access to resources and social participation in decision making [29]. HSD is inherently political because health decisions often depend on political decision makers and legislators.

Good leadership can positively impact HS performance. The lack of a visionary and prospective leadership is reflected in the lack of serious planning and the failure to implement existing programs. Just like other social institutions, HCS is affected by negative public values (corruption, greed and lack of professionalism). Chad needs more selfless leaders, nation-centered individuals who are real servants of their people. Servant leaders focus on serving others rather than themselves. They are visionaries who are committed to stewardship and to the growth of their communities, rather than to improving their financial status and political influence [30]. While there is life-giving potential in the ideal of selfless leadership, self-interested leadership undermines patriotic values; those values that empower individuals to work for the progress of the country. Inversely, good leadership is all about opposing greed to promote an organizational culture that gives pre-eminence to the national interest. Visionary leadership anticipates tools needed for decision-making and management for public infrastructures. The absence of vision is perceptible, for example, through the absence of standards for managing infrastructures and equipment and by the lack of an updated sanitary card which could be used as a tool for public health decision making.

Conceptual weaknesses of policy frameworks and regulations limit the implementation of the National Health Policy (NHP). From the perspective of the RH, effective implementation is absolutely critical. Without the concrete implementation of the NHP, both progressive realization of and effective access to care, which are two important components of the RH, remain unfulfilled wishes [26]. Program implementation often needs research for effective realization. Health interventions such as diagnostics, access to care and public health programs are often not grounded in prior research. For example, implementation research to discern the socio-historical and cultural roots of both the under-utilization of healthcare services and the failures of previous public health interventions is rarely launched. Implementation research in poor or rural areas should have a social action component, so as to address both disease etiology and the social roots of implementation failure.

Good governance is critical to public health because it shapes the institutional and legal environment of HS. Governance determines sectoral and intersectoral dialogue, shapes the direction and coordination of all activities of the Ministry of Public Health (MPH) and influences the definition of priorities and the development of rules to promote fairness in the distribution of available resources. In Chad, financial governance is weak because of repeated suspensions of the financial execution of budgeted expenditure and because non-compliance with the legislation governing the management of public finances prevents a timely execution of programs. Governance is weakened when spending authorizations are issued with delays that hinder the implementation of programs at the regional level [12]. The insufficiency of resources allocated to the health sector is often due to poor planning and management of data at all levels, excessive centralization of operating funds, less involvement of regional health commissioners in the MPH’s process of budgeting, insufficient coordination between the state and its financial partners, and the mobilization of aid on the basis of national programs and cooperation projects with their own management mechanisms [12].

The technical governance of HS is also poor. The sector lacks clear prospects supported by a well-thought-out system of coordination. The actual state of HS and the poor performance of healthcare system reflect a lack of coordination at and between the three levels of the healthcare pyramid. The same lack of coordination is also perceptible at the regional level, due to the lack of both resources and sustained support from the MPH. The MPH needs to improve its supervision capabilities by developing effective coordination mechanisms and evaluation tools to monitor activities and resources in the health sector. Beyond the power of the MPH, inter-sectoral collaboration ensured by coordinated governmental policy may significantly contribute to health system development. Infrastructures, for example, depend on another ministerial department beyond the control of the MPH. To improve facilities in the health sector, the MPH and the ministry of infrastructures have to collaborate. Similarly, success in reducing maternal and infant mortality, improving access to care and environmental surveillance, and addressing barriers to good health cannot be achieved solely by the coordination of the MPH. There is need for an integrated governmental policy that creates areas of intersection between the spheres of intervention of each ministry. There should be a special unit within the government that could constantly monitor and refine such concerted endeavors.

Improving ownership by reducing donor dependency and vertical programming needs strong leadership. National HS receive resources and technical assistance from many different non-governmental organizations (NGOs), donor programs and projects with varied priorities and demands, placing pressure on the MPH to favor vertical programming to respond to short-term public health challenges [31]. The three-disease program (malaria, tuberculosis and AIDS) subsidized by the Global Fund is an example of a vertical program which is being carried out right now in Chad. The verticality of this program lies in the fact that its implementation takes place outside of integrated HS. There is increasing debate about whether this program is strengthening or weakening the Chadian HS. Vertical programming is often needed when a country does not have enough resources to face a public health emergency. The three-disease program has improved the primary healthcare system, has improved disease surveillance through parallel monitoring and has begin to incorporate mother-to-child transmission strategy into health services. Even though these program and others have achieved important gains, consistently ensuring resources for development of HS has not been easy [32]. Vertical interventions draw off resources from HS and can jeopardize progress toward the long-term goal of an effective health system [26]. To consolidate the good practices gained from the three-disease program, reduce the fragmentation of HS and minimize the duplication of services, the integration of the three-disease program within the healthcare system needs to be done.

An influx of NGOs in the country now exacerbates the fragmentation of national HS through their project activities, which divert financial and human resources away from the public sector. NGOs often create structures parallel to government services, structures that do not ensure a sustained provision of care, especially to isolated populations. At the same time, we should appreciate the contribution of NGOs to the health sector, especially their effort to build health facilities in marginalized areas. NGOs need to be challenged by the government to commit to HSD. The government may, for example, ask every NGO to sign a code of conduct that expresses its commitment to work for unified HS. Enforcing the Paris Declaration on Aid Effectiveness may help achieve such a goal. This Declaration challenges donors to support and to scale up effective programs and projects by strengthening the host country’s development strategies and HS operational procedures [33].

### Access to care and the centrality of the primary healthcare system

Primary care helps prevent illness and death, regardless of whether the care is characterized by supply of primary care physicians, a relationship with a source of primary care, or the receipt of important features of primary care. The evidence also shows that primary care (in contrast to specialty care) is associated with a more equitable distribution of health in populations. The means by which primary care improves health have been identified, thus suggesting ways to improve overall health and reduce differences in health across major population subgroups.

Health worker shortages and weak HS have led to a lack of preventive and curative health care services and health promotion programs in various parts of the country, making it difficult for the country to improve health indicators. The HS suffer from insufficient financial and human resources, limited institutional capacity and infrastructure, weak health information systems, lack of comprehensiveness, embedded inequity and discrimination in availability of services and a lack of management capacity-building [34]. Financial barriers to care challenge the country and its partners to develop new approaches to healthcare financing, organization and delivery, since out-of-pocket payment has failed as a health-financing policy. The lack of financial protection for costs of healthcare pushes many households below the poverty threshold, while others cannot seek care due to lack of funds [35].

The Minimal Package Activities (MPA) and Complementary Package Activities (CPA) are two important elements of the NHP, constituting the bedrocks of primary healthcare systems (PHCS) as means for ensuring an effective delivery of the minimal package of health services to all. The NHP prescribes that the MPA and CPA should ensure integrated, comprehensive and continued care. However, such an aim is not being fully achieved because supply is very low and service delivery is incomplete, with significant differences from one district or region to another [12]. Access differential rates are due to sociocultural, economic, geographic and structural reasons. Maternal mortality results from the lack of resources, lack of access to emergency and obstetric care, and lack of qualified staff. Drug supply shortage and a dysfunctional referral system also contribute to maternal mortality. Shortcomings in vaccination programs are related to irregular supply of vaccines, inefficient delivery strategies and inadequate data management at the district level. Improving the primary healthcare system, rather than specialized healthcare institutions for maternal and child health, is critical for the success of child and maternal health interventions. Specialized care is important, but it cannot supersede basic primary healthcare (PHC) for children and mothers. Improved PHC coverage is threatened by a growing tendency to hospital-centrism [36].

Hospital-centrism can be defined as “disproportionate focus on specialist and tertiary care” [36] to the detriment of the continued improvement of structures of PHC that may contribute to significant reductions of mortality, morbidity and preventable diseases. Recently, many specialized healthcare institutions have been built, requiring high-tech equipment and highly trained specialists that the state cannot always afford to provide in a sustainable manner. Worldwide, the forces driving the disproportionate focus on tertiary care include professional interests and the economic interests of the medical technology and pharmaceutical industries [36]. Over-medicalization of the healthcare system (HCS) may not yield positive results in public-health terms because such a tendency may drive attention away from the attention accorded to marginalized groups, preventive care and health promotion activities. In a country where access to basic care remains a major challenge, hospital-centrism represents an inefficient way of using scarce resources and a real source of health inequity, because it presupposes that, in order to access care or health information, people should visit a healthcare institution.

To improve access to healthcare, strategies should be developed to improve the effectiveness of interventions and HCS should provide financial incentives for those who use services. Reliance on community mobilization techniques can also be instrumental in improving the use of health services, especially the use of local healthcare centers and district hospitals. How can the use of health services be improved without addressing financial, economic and physical barriers to care? The expansion of close-to-people services can reduce physical barriers, while financial barriers may be removed at point of service through increased prepayment and increased responsiveness of providers, as is being done through pay-for-performance approaches at the healthcare center level. Service delivery demands a sufficient workforce. Addressing the shortage and poor distribution of adequately trained staff at PHC level can be done by increasing the number of healthcare workers, implementing task shifting, and increasing allowances for those who work in hard-to-reach areas [37]. The implementation of these measures will increase HS performance and staff motivation. Increasing pay and improving supervision can be ways to address staff motivation.

### Accountability and social inclusion

Accountability is more than ensuring that health funds have been properly used. It includes mechanisms ensuring health systems development, community participation and inclusion of disadvantaged individuals and communities.

### Accountability mechanisms and transparency

Accountability is also concerned with monitoring conduct, performance and outcomes of institutions and individuals involved in HCS management [26]. HS lack “transparent and effective mechanisms of accountability to understand how those with responsibilities towards the health system have discharged their duties” [26]. Even when these mechanisms exist, they are not accessible to the public. Very often these mechanisms are imposed by donors, international institutions or NGOs. In the context of an overall lack of institutional transparency, even international NGOs do not always abide by the moral imperative of accountability. Yet, “there must be accessible, transparent and effective mechanisms of accountability to understand how those with responsibilities towards the health system have discharged their duties” [26]. A rights-based approach to health information systems can create venues for accountability and can unveil health inequalities [36]. However, health information remains insufficient to support basic analysis of socioeconomic inequalities, from which analysis policies to reduce health inequalities can be designed.

The office of health commissioners, democratically elected health councils, public hearings, patients’ committees, impact assessments and judicial proceedings are mechanisms of accountability often used in countries across the globe [26]. These mechanisms have not yet been adopted by policymakers and legislators in Chad. As a social entity, how can HS abide by the ethical requirements of democratic accountability when other social institutions do not? Nevertheless, to ensure an effective functioning of the healthcare system (HCS), the Ministry of Public Health (MPH) can rely on a wide range of accountability mechanisms that have proven to guarantee the transparency and effectiveness of health policy decisions, health sector programs and financial management. Accountability demands that the MPH coordinate and monitor resource allocation efficiently and equitably, according to well-defined priorities and criteria. Concerning the governance of the health aid structure, there is a need to improve and harmonize coordination mechanisms among donors and partners at all levels of the HCS to improve health services and reach out to the most vulnerable population groups. Uncoordinated health aid has contributed to the fragmentation of HS and to duplication of services and monitoring mechanisms. The MPH need to design a legal and policy framework to guide health aid coordination.

### Inclusion of disadvantaged individuals, communities and populations

Over the last 15 years, Chad has enjoyed a certain level of economic growth, with oil drilling and international money destined to humanitarian work. The private health sector has flourished. Often influenced by the weight of disease-specific interventions and the poor financial state of HS, failures to regulate the healthcare sector adequately have led to the mushrooming of small-scale profit-based healthcare institutions offered by a multitude of different independent providers who do not always abide by professional standards [37]. Even though decisions have been made to provide free access to emergency care, as well as to maternal and infant care, as a way to reduce infant and maternal mortality, healthcare in Chad remains a commodity. Patients are often seen as mere clients, while healthcare is seen as a good like any other good. Commoditization of healthcare not only compromises the quality of care but also impedes access to care. Most well-off people go to quality private providers to avoid the bureaucracy and inefficiency of the public system. Even in public hospitals, there are separate admittance rooms for the well-off and the poor.

The engagement of the private sector remains a topic of considerable controversy, seen by some as inviting the privatization of health care and making it a commodity and by others as a call for building a primary healthcare system that includes or restricts the role of the private sector [38]. However, realistically, when the capacity of the public sector is limited, there is a concentration of human resources in the private sector. Efforts to seek a combination of public and private provision of services may be seen as a pragmatic and realistic response to the public’s health needs. However, in Chad, private providers cannot guarantee healthcare access to the poor, because they receive no subsidies from the government. Hence, “we posit that within any mechanism of care delivery, the government is responsible for ensuring that the poor get the treatment that is their right” [23]. International institutions may preferentially accompany the public sector to build its capacity to deliver services and, simultaneously, work with communities and civil society to hold government accountable [23]. Strengthening the capacity of the formal private sector may look like a war on the poor. However, outreach campaigns to improve drug accessibility for certain types of diseases to the poor by training drug sellers to good delivery, practices as has been done on the Kenyan coast for artemisinin-based combination therapies against malaria can be seen as a useful intervention [39].

The number of public healthcare institutions has significantly increased under the leadership of the current head of state. Yet, health indicators have not significantly improved. The geography of health inequality may explain partially why there is hardly any improvement in public health. Health differentials raise important issues of social justice. Social justice refers to the ethical imperative of enabling all individuals and specific groups to seek and to receive services that are commensurate with their needs, while providing them with the basic dimensions of justice. Just access to care and to other dimensions of well-being cannot be achieved if the healthcare system is not responsive to local demands. The concept of responsiveness refers to the human rights imperative imposed on HS to be sensitive to people’s aspirations, needs and demands by offering adequate support and services. The principles of non-discrimination, equality and human dignity are critical to the way HS responds to people’s health needs.

The principles of equality and non-discrimination are among the most fundamental principles of human rights thinking and practice. Discrimination occurs when a person is treated unjustly on the basis of his/her “race, color, sex, language, religion, political or other opinion, national or social origin, property, disability, birth or other status” [40]. The magnitude of public health challenges and of access barriers to care for the poor demand an effective intervention by the government as a way to protect the well-being of those who are already marginalized by social forces. The prioritizing of expensive curative health services, instead of preventive and primary healthcare, is considered to be “Inappropriate health resource allocation [that] can lead to discrimination that may not be overt” [9]. Nondiscrimination also refers to the fact that the realization of the RH includes participatory decision making by the people, especially the most vulnerable, to whom priority should be accorded while making health planning and resource allocation decisions. In Chad, the HS essentially discriminates on the basis of sex, age, social status and geographic location.

Even when health-related facilities and services are available to all, marginalized population groups find it hard to access them. Women’s and children’s access to antiretroviral treatment (ART), for example, is constrained by many factors that place their lives at stake. Even though women represented 65% people living with AIDS in 2009, their access to antiretroviral drugs (ARVs) was limited compared to that of men [12]. Just two examples show that children’s health needs are not always given serious consideration. Only 9% of children living with HIV were on ARVs in 2009, against 47% of adults. In addition, activities for prevention of mother-to-child transmission (PMTCT) have not evolved significantly so as to eradicate mother-to-child transmission. The extension of PMTCT was limited to 120 sites in late 2011, with a geographical coverage of 13%, and ARVs reached only 11% of pregnant women in 2012 [37]. So far, only five health districts integrate PMTCT activities in antenatal care mobile strategies [12].

The rural–urban divide is another source of discrimination rooted in the overall culture of exploitation of remote parts of the country and poor decisions concerning allocation of resources. Rural centers are centers of agricultural production, but in return they do not get much from the government. In addition to this reason, resource allocation does not always follow the principle of equity, meaning resources would be distributed according to public health needs. Three examples illustrate this divide clearly: the distance between homes and healthcare institutions, access to medical technology and the distribution of physicians across the country.

The National Health Policy (NHP) defines geographical accessibility of care as the ability to access care within a distance of 10 km [14]. Healthcare institutions are close to residential areas in urban centers, but are lacking in rural areas. Only 51.80% of the entire population lives at a distance less than or equal to 10 km from a healthcare institution [41]. More than 25% of healthcare centers are located between 2 and 6 hours walk from the nearest reference hospital. This distance is greater for the rural population than for the urban, and it varies from one region to another. The distance between healthcare institutions and the nearest referral hospitals is a major determinant of survival when serious complications occur.

The rural–urban divide is also exemplified by the relationship between women’s education and infant malnutrition and mortality rates. Although economic deprivation is associated with a lower level of education and literacy among women (67%) as compared to men (41%) all over the country, women’s education level varies more significantly by place of residence. Almost 50% of urban women are literate, against 7.7% in the countryside. Education level predictably limits women’s access to health information. The infant mortality rate and child malnutrition are closely related to the mother’s education level: 128 per 1000 for children whose mothers’ instruction level is equal to or higher than secondary school and 191 per 1000 for children whose mothers attended only primary school [10]. The proportion of underweight children is 35% for those whose mothers have no education, against 15% for those whose mothers have secondary education. Considering that women’s education level is higher in urban than in rural areas, it can be inferred that high malnutrition and mortality rates affect rural children more than urban ones. Even though malnutrition is a widespread phenomenon in Chad, rural children (41%) are more affected than urban ones (31%). About 30% of children are underweight, including 13% who are severely underweight. Again, rural children are more affected (33%) than urban ones (22%). About 16% of children are severely or moderately emaciated. A malnourished child has high chances of contracting diseases, and a sick child is also more likely to become malnourished [13]. Part of the reason malnutrition is recurrent is because, even when harvests are plentiful, poor families are forced to sell much of their harvest to repay debts incurred during the dry season, selling often at low prices due to high market supply. With reduced stocks to run through the lean period, they are again forced into debt.

Relevant to the realization of the RH is the distribution of health professionals across regions. Health professionals are unevenly distributed, with a high concentration of the more qualified ones in urban centers, especially in N’djaména. There is a deficit of doctors in most regions, while there is a high concentration of them in N’djaména, whose population represents only 9.71% of the entire population. This distribution is not only uneven but disproportionate compared to regional health needs [12]. More than half of midwives and doctors practice in N’djaména. The mobility of health professionals is a recurring problem that undermines efforts to implement the NHP. Legislation such as incentives for those working in rural areas can encourage health professionals to willingly accept to work in remote districts.

Relevant to health justice is the right to “enjoy the benefits of scientific progress and its implications” [3]. This right is not achieved when medical technologies are concentrated in regional hospitals located in major cities. This right can also be cited to challenge the low achievement of universal access to ART and the deficient rate of vaccine coverage. States have an obligation to prohibit and eliminate discrimination on all grounds and to ensure the equality of all citizens in relation to access to healthcare services and the underlying determinants of health. Equality refers to the assumption that each person matters; everyone deserves to be treated with dignity. Claims on behalf of human dignity express the conviction that people are owed certain forms of treatment simply by virtue of their humanity. Human dignity is an inherent quality and not an acquired one; it is a kind of intrinsic worth that belongs equally to all human beings as such [41]. Autonomy, reasonability or the fact of being created in the likeness of God are often appealed to as the substantive foundation of human dignity. The RH reflects a particular specification of certain minimum preconditions for a life of dignity. Respect for human dignity is the tenet from which all human rights flow. Disadvantaged individuals and populations are entitled to special protection. Their marginalization by the healthcare system (HCS) is rooted in structural inequalities that shape the social landscape. To achieve equity in service provision, outreach programs that target the marginalized must be in place, to ensure that they enjoy the same access as those who are more advantaged.

There has been an ongoing debate about universal coverage of ART in Chad. The successes and failures of the antiretroviral treatment universal access policy have taught us a great deal. We can no longer minimize social and other barriers to access [42]. For example, “other out-of-pocket expenditures can present a significant barrier to people gaining full access to HIV/AIDs treatment and care services. Quite often, the free ARV package does not cover diagnostics, formal or informal fees, transport to and from the health service, and so forth, which are strong risk factor for mortality” [43]. Universal coverage is a sure way of improving equity in access to health services. Achieving universal coverage entails securing equitable access to essential health services through proper planning, resource allocation and implementation processes that improve the use of health services by poor and vulnerable groups, taking gender and age into account [44]. There are compelling evidentiary proofs for reconsidering the importance of PHCS for health promotion as an approach that promotes equitable health and human development. In addition to supporting some vertical programs, the international community needs to fund activities related to the development of HS, especially the primary healthcare system (PHCS) to allow universal access to Minimum Package of Activities and Complementary Package of Activities by all. Chad does not have to money to undertake such an important initiative. Sustained funding for specific diseases that need long-term monitoring and intervention remains important, but underfunded HS will fail to integrate the gains from such interventions. The RH movement can be the backbone of initiatives aiming at designing a new HCS that is inclusive and reluctant to allow unjustified inequalities, lack of professionalism and the exclusion of local communities from the sphere of decision making. The three-disease program has set the momentum to move from a still hesitant step to a more aggressive and systematic management of synergies between vertical programs and health systems strengthening in Chad, with a specific priority accorded to PHCS.

### Community agency and social participation

The HCS is highly bureaucratic, with weak and overly centralized planning and decision-making systems that do not allow subsidiary bodies to participate in policy making. Decentralized decision making is needed to include input from regional and district levels. However, partnership between government and civic organizations, which would ensure accountability and transparency in health-sector management through participation in planning and service oversight, is lacking. Government agents are absolute decision makers in and managers of the entire healthcare system (HCS).

Public health has a long tradition of recognizing social participation as an integral part of health promotion. Realizing the RH implies providing individuals and communities with an authentic voice in decisions defining and affecting their well-being [45]. Social participation is an important asset to primary healthcare (PHC) initiatives because, as the primary beneficiary [26], the community is well-placed to promote its own welfare. Social participation results in greater responsiveness on the part of community members. Citizens who adequately know their rights and responsibilities are assets in ensuring the development of HS for the good of all. A wider participation in the decision making processes creates local entities that protect public interests, and, thus, this participation promotes accountability. In Chad, even an empowered community cannot hold the government accountable for the quality, equity and effectiveness of HCS because the RH has not been substantially introduced into the penal code with all the enforcing mechanisms. Due its instrumentality, the RH should normally be considered as guaranteeing a fair trial in the court system because it highlight the state’s legally-binding obligations to public health, especially when reforms or policies that may hinder access to health services by disadvantaged individuals and groups are imposed on policymakers by national or international forces [46]. Even though the RH has been incorporated in the constitution in Articles 17, 18 and 26 [5], its introduction in the penal code emphasizes personal or family health and not public health in a systematic manner. The RH has not been framed in such a way that a community or a particular social group can file a complaint against the state. A constitutional and penal code reform is needed to provide the country with legal arrangements that can protect and promote public health. More laws that give people the right to challenge government action in the field of public health are needed.

Since 1994, community participation has gradually been introduced into the HCS. Law No. 019/PR/99 of 10 December 1999 established community participation in financing, planning, management and evaluation of health resources [47]. Although community participation enjoys legal recognition, healthcare center committees (HCCs) do not have legal status. HCCs are recognized simply as entities for improving management and service delivery in healthcare centers. A reform of the national public health law is needed to provide a legal status to HCCs at the PHC level. The activism of HCCs creates a dialogue between the community and the healthcare center. This dialogical endeavor brings about a constant flow of information and feedback instrumental in health promotion. Community health workers (CHW) are chosen by the HCC to participate in service delivery. CHW have recently been involved in the distribution of Mectizan and mosquito nets and in social mobilization, immunization campaigns and HIV prevention campaigns [12]. Unfortunately, no study has been launched to assess the functionality of CHW in order to improve their usefulness. So far, where they are well-established, traditional birth attendants and other CHW have been successful in increasing the number of assisted deliveries by convincing pregnant women to attend antenatal and postnatal consultations [10].

## Conclusion

Relying on the right to health (RH) as the bedrock for health systems development (HSD) brings to the forefront the structural, ethical and technical limitations of HS in Chad. A fragmented, understaffed, technocrat-based and underfunded HS cannot realize the RH. Chad has one of the highest infant and maternal mortality rates of the world, poor access to primary healthcare, a highly hazardous environment, deficiency in coordination, and a permissive epidemiologic surveillance system, coupled with a lack of public values and deontological arrangements that determine the functioning of social institutions. Given all these negative factors, achieving the RH in Chad remains a mere dream. Addressing progressively the limitations of all the components of HS and providing, through various mechanisms, other goods which together protect health appears to be the way ahead to ensure people’s well-being. The realization of RH requires a radical policy shift rooted in inter-sectoral collaboration, community participation, public accountability and transparency, and social justice in a country where democracy is still growing. In such a context, HS strengthening requires a focus not only on specific strategic interventions and policy solutions, but also on the creation of an environment that supports good practices and sound public values. HS strengthening must thus be seen as a long-term endeavor that involves complex systems and requires carefully shaped intervention in many arenas under the leadership of the Ministry of Public Health.

## Abbreviations

RH: Right to health
HS: Health systems
HSD: Health systems development
PHC: Primary healthcare
UDHR: Universal Declaration of Human Rights
WHO: World Health Organization
HCS: Healthcare system
AIDS: Acquired Immuno-deficient Syndrome
HIV: Humane Immuno-deficient Virus
MPA: Minimum Package of Activities
CPA: Complementary Package of Activities
ANC: Antenatal Care
MPH: Ministry of Public Health
NHP: National Health Policy
NGO: Non-governmental Organization
ARV: Antiretroviral drugs
ART: Antiretroviral Treatment
PMTCT: Prevention of Mother to Child Transmission
HCC: Healthcare Center Committee
CHW: Community Health Workers
